# Remdesivir and Cyclosporine Synergistically Inhibit the Human Coronaviruses OC43 and SARS-CoV-2

**DOI:** 10.3389/fphar.2021.706901

**Published:** 2021-08-13

**Authors:** Hsing-Yu Hsu, Cheng-Wei Yang, Yue-Zhi Lee, Yi-Ling Lin, Sui-Yuan Chang, Ruey-Bing Yang, Jian-Jong Liang, Tai-Ling Chao, Chun-Che Liao, Han-Chieh Kao, Szu-Huei Wu, Jang-Yang Chang, Huey-Kang Sytwu, Chiung-Tong Chen, Shiow-Ju Lee

**Affiliations:** ^1^ Institute of Biotechnology and Pharmaceutical Research, National Health Research Institutes, Miaoli, Taiwan; ^2^ Institute of Biomedical Sciences, Academia Sinica, Taipei, Taiwan; ^3^ Institute of Clinical Laboratory Sciences and Medical Biotechnology, College of Medicine, National Taiwan University, Taipei, Taiwan; ^4^ National Institute of Infectious Diseases and Vaccinology, National Health Research Institutes, Miaoli, Taiwan

**Keywords:** COVID-19, cyclosporine, IL-6, IL-8, OC43, remdesivir, SARS-CoV-2, synergistic

## Abstract

Remdesivir, a prodrug targeting RNA-dependent-RNA-polymerase, and cyclosporine, a calcineurin inhibitor, individually exerted inhibitory activity against human coronavirus OC43 (HCoV-OC43) in HCT-8 and MRC-5 cells at EC_50_ values of 96 ± 34 ∼ 85 ± 23 nM and 2,920 ± 364 ∼ 4,419 ± 490 nM, respectively. When combined, these two drugs synergistically inhibited HCoV-OC43 in both HCT-8 and MRC-5 cells assayed by immunofluorescence assay (IFA). Remdesivir and cyclosporine also separately reduced IL-6 production induced by HCoV-OC43 in human lung fibroblasts MRC-5 cells with EC_50_ values of 224 ± 53 nM and 1,292 ± 352 nM, respectively; and synergistically reduced it when combined. Similar trends were observed for SARS-CoV-2, which were 1) separately inhibited by remdesivir and cyclosporine with respective EC_50_ values of 3,962 ± 303 nM and 7,213 ± 143 nM by IFA, and 291 ± 91 nM and 6,767 ± 1,827 nM by a plaque-formation assay; and 2) synergistically inhibited by their combination, again by IFA and plaque-formation assay. Collectively, these results suggest that the combination of remdesivir and cyclosporine merits further study as a possible treatment for COVID-19 complexed with a cytokine storm.

## Introduction

COVID-19 has affected more than 176.887 million people in 194 countries and caused over 3.84 million deaths (as of 2021-06-18 https://www.cdc.gov.tw/) since its emergence at the end of 2019. This disease is caused by infection with Severe Acute Respiratory Syndrome Coronavirus 2 (SARS-CoV-2), and disease progression is usually complexed with a cytokine storm and/or organ dysfunction (McElvaney et al., 2020).

Interleukin-6 (IL-6) and other cytokines are increased in patients with COVID-19, and IL-6 was also found to be a hallmark predictor for COVID-19 progression. (Chen et al., 2020; McElvaney et al., 2020; Yang et al., 2020). Tocilizumab, a monoclonal antibody against IL-6 receptor (IL6-R) is an immunosuppressive drug originally developed for the treatment of rheumatoid arthritis (Scott, 2017) and systemic juvenile idiopathic arthritis (De Benedetti et al., 2012), but is currently under development as an alternative therapy for COVID-19 patients who are at risk of a cytokine storm (Rosas et al., 2021). Recently, preliminarily data showed tocilizumab to be associated with significant clinical improvement and a reduction in mortality (Klopfenstein et al., 2020; Toniati et al., 2020; Xu et al., 2020). Therefore, mitigation of the cytokine storm that occurs in COVID-19 patients is a sound therapeutic strategy.

Remdesivir targets SARS-CoV-2 itself, inhibiting viral RNA-dependent-RNA-polymerase (RdRp) (Gordon et al., 2020; Pruijssers et al., 2020) and was the first approved treatment for COVID-19, although its efficacy is restricted to a shortening in the time to recovery of hospitalized patients (Beigel et al., 2020). Nonetheless, COVID-19 symptoms and mortality can be ameliorated by anti-inflammatory or immunomodulatory agents (Fatima et al., 2020; Tomazini et al., 2020). Thus, the combination treatment of remdesivir with existing anti-inflammatory or immunomodulatory agents may exert an especially therapeutic effect COVID-19 patients and merits further exploration.

The repurposing of existing drugs to take advantage of their known safety profiles and associated commercial supply chains is an important strategy to expedite the discovery of effective and safe COVID-19 treatments. Moreover, the combination of existing drugs with remdesivir may also improve its effectiveness. SARS-CoV-2 related experiments are regulated and can only be performed in biosafety-level-3 (BSL-3) or higher laboratories, but the number, resources, and capacities of such laboratories are very limited. Therefore, we used the alternative coronaviruses swine transmissible gastroenteritis virus and human flu coronavirus OC43 (HCoV-OC43) as SARS-CoV-2 surrogates, to assay drugs for anti-viral activity prior to testing with SARS-CoV-2 itself. About 230 prescription drugs covered by Taiwan Health Insurance were screened, cyclosporine, an immunosuppressant widely used to prevent organ transplant rejection (Beauchesne et al., 2007; Wu et al., 2018), was found to significantly reduce infection by coronaviruses. Cyclosporine was first isolated from the fungus *Tolypocladium inflatum* (*T. inflatum*) in soil samples by scientists from the Norwegian Sandoz Pharmaceutical Company in 1969 (Beauchesne et al., 2007) (Yang et al., 2018). It is a lactam comprising 11 amino acids (including a D-amino acid) and is synthesized by ciclosporin synthetase, in contrast to most peptides that are synthesized by the ribosomes (Yang et al., 2018). Cyclosporine suppresses the activity of the immune system by inhibiting the activity and growth of T cells (Liddicoat and Lavelle, 2019) as well as IL-6 production (Stephanou et al., 1992; Iacono et al., 1997; Golling et al., 2009). NF-κB suppression by cyclosporine inhibits the production of proinflammatory cytokines (Meyer et al., 1997; Du et al., 2009; Jerkins et al., 2020).

Herein, we examine the inhibitory activities of combined treatments of remdesivir and cyclosporine against IL-6 cytokine production and the HCoV-OC43 and SARS-CoV-2. The results obtained suggest a potential regimen for combating COVID-19 complexed with a cytokine storm.

## Materials and Methods

### Chemicals and Antibodies

DMSO (D1435, ≥99.5%), crystal violet (C0775, dye content ≥90%), and methylcellulose (#M0387) were purchased from Sigma-Aldrich (St. Louis, MO, United States); remdesivir (GS-5734) (S8932, 99.3%, HPLC) and cyclosporine A (cyclosporine) (S2286, 99.6%, HPLC) from Selleckchem (Houston, TX, United States); Goat anti-human IgG-Alexa Fluor 488 (A11013), Hochest 333258 (H3569), and DAPI (D1306) from Invitrogen (Thermo Fisher Scientific, Waltham, MA, United States); and 10% formaldehyde solution from Marcon™ Chemicals (#H121-08). The antibody against nucleocapsid (N) protein of HCoV-OC43 (Mab9013) was purchased from Merck Millipore (Burlington, MA, United States), and fluorescein isothiocyanate (FITC)-conjugated anti-mouse immunoglobulin (#55499) from MP Biomedicals (Irvine, CA, United States). Anti-SARS-CoV-2 N protein antibodies were provided by Dr An-Suei Yang of the Genomics Research Center, Academia Sinica.

### Cell Lines, Virus, Western Analysis and Immunofluorescence Assay for HCoV-OC43

Human colon adenocarcinoma cell line HCT-8 (ATCC® CCL-244™) and human lung fibroblasts cell line MRC-5 (ATCC® CCL-171™) were obtained from American Type Culture Collection (ATCC), passaged within 6 months of receipt, and established as stocks in the cell bank at an early passage, to ensure cell line-specific characteristics. The subsequent 4th to 20th passages of HCT-8 cells and 7th to 16th passages of MRC-5 cells were used in this study. Heat inactivated premium grade fetal bovine serum (FBS) from VWR Life Science Seradigm (Radnor, PA, United States) was used to culture MRC-5 cells and FBS from Biological Industries Inc. (Cromwell, CT, United States) was used for the HCT-8 cell culture to obtain optimal culture conditions. HCoV-OC43 (ATCC® VR1558™) was grown and propagated in HCT-8 cells cultured with DMEM and 2% FBS. Time course experiments for the detection of HCoV-OC43 N protein with the antibody Mab9013, western blot [as described (Yang et al., 2020)], and IFA were performed with the samples at the indicated time points. For compound treatment studies, HCT-8 or MRC-5 cells were seeded in 96-well plates and then cultured in DMEM or MEM medium containing 2% FBS, respectively. Cells were pretreated with serial dilutions of remdesivir and cyclosporine at the indicated concentrations for 0.5 h prior to HCoV-OC43 infection at an MOI of 0.05. At 30 h.p.i., the resulting adherent cells were then fixed with 4% formaldehyde, permeabilized with 100% methanol, and subsequently subjected to IFA analyses with an antibody against HCoV-OC43 N protein (Mab9013) and FITC-conjugated anti-mouse immunoglobulin (#55499) (green), and the EC_50_ values determined as described (Yang et al., 2020). CC_50_ values were determined by staining nuclei blue with Hoechst dye and then determining relative cell viability using the number of nuclei in the vehicle control as 100%. Fluorescent signals were detected and quantified using the ImageXpress Micro XLS Widefield High-Content Analysis System (Molecular Device). The fluorescent signal was normalized with cell viability to calculate the infection rate that no compound treatment was set at 100%. Synergy scores for combined treatments were calculated using SynergyFinder (https://synergyfinder.fimm.fi/).

### Cell Line, Virus, IFA, and Plaque Assay for SARS-CoV-2

For IFA used in the SARS-CoV-2 study, Vero E6 cells (BCRC number: 60476; derived from ATCC CRL-1586) were passaged within 6 months of receipt and the 4th–15th passages used. Cells were pretreated with each compound at the indicated concentration for 1 h at 37°C and then adsorbed with SARS-CoV-2 (TCDC#4) (sequence available on the GISAID website) at 100 PFU (MOI = 0.01) for 1 h at 37°C as described (Yang et al., 2020). The virus-containing supernatants were removed and then fresh medium containing each compound at the indicated concentrations added to the cells. After incubation for 1 day, cells were fixed and immunostained with anti-SARS-CoV-2 N protein antibody (provided by Dr An-Suei Yang) plus anti-human IgG- Alexa Fluor 488 (A11013, Invitrogen) (green). Nuclei were counter-stained with DAPI (blue); the number of nuclei in vehicle control (no compound treatment) was defined as 100%; and then relative cell viability and CC_50_ values were determined. The fluorescent signal was quantified by high-content imaging and the infection rate and EC_50_ values calculated using normalized values with respective cell viability; the infection rate was defined to be 100% for samples where no compound was added. For combined treatments, the synergy scores were calculated by SynergyFinder (https://synergyfinder.fimm.fi/).

Plaque assays for SARS-CoV-2 were performed as described (Yang et al., 2020). Briefly, Vero E6 cells were seeded 1 day before infection with SARS-CoV-2 for 1 h at 37°C. Subsequently, viruses were removed and the infected cells were covered with overlay media containing 1% methylcellulose (Sigma, cat #M0387) and the test compounds at the indicated concentrations. At 5–7 days post infection, cells were fixed overnight, the overlay media was removed, the resulting cells were stained with crystal violet, and the plaques were counted. Plaque numbers were corrected by plaque size: for a plaque size 50% smaller than the vehicle control, the plaque counted for 0.5 plaque; for a plaque size 75% smaller than the vehicle control, the plaque counted for 0.25 plaque. The percentage of inhibition was calculated as [1–(VD/VC)] × 100%, where VD and VC refer to the virus titer in the presence and absence of the test compound, respectively. The cell viability was determined as described (Kuo et al., 2021) by measurement of relative alkaline phosphatase activity; viability was defined to be 100% for samples where no compounded was added.

### Interleukin 6 Cytokine Measurement

IL-6 cytokine levels in the culture supernatants of HCoV-OC43 infected MRC-5 cells at 30 h.p.i. were detected and quantified. Test supernatants were diluted to the requisite concentrations using human IL-6 enzyme-linked immunosorbent assay kits (ARG80110) from arigo Biolaboratories Corp. (Hsinchu, Taiwan) per the manufacturer’s recommendations.

### Drug Combination Study

Viral inhibition by remdesivir, cyclosporine, and their combinations as measured by IFA or plaque assay was assessed by a drug dose-response matrix. Average synergy scores were obtained via the online tool SynergyFinder (https://synergyfinder.fimm.fi/). ZIP synergy scores were calculated and plotted from three independent experiments. The interaction between two drugs was considered antagonistic for ZIP synergy scores of less than −10; likely to be additional for scores between −10 and 10; and synergistic for ZIP synergy scores of greater than 10.

### Statistical Analysis

The statistical significance between the two groups was evaluated using one-way ANOVA followed by Tukey’s multiple comparison test in GraphPad Prism (version 8) software; The two-tailed unpaired Student’s t test was used evaluated the dose effect of single drug treatment on IL-6 production. * and ** denote statistical significance of *p* < 0.05, and *p* < 0.01 respectively.

## Results

### Inhibitory Effects of Remdesivir and Cyclosporine, Alone and in Combination, on HCoV-OC43 in Human HCT-8 Colorectal Carcinoma Cells and Human MRC-5 Fetal Lung Fibroblast Cells (IFA)

**FIGURE 1:**
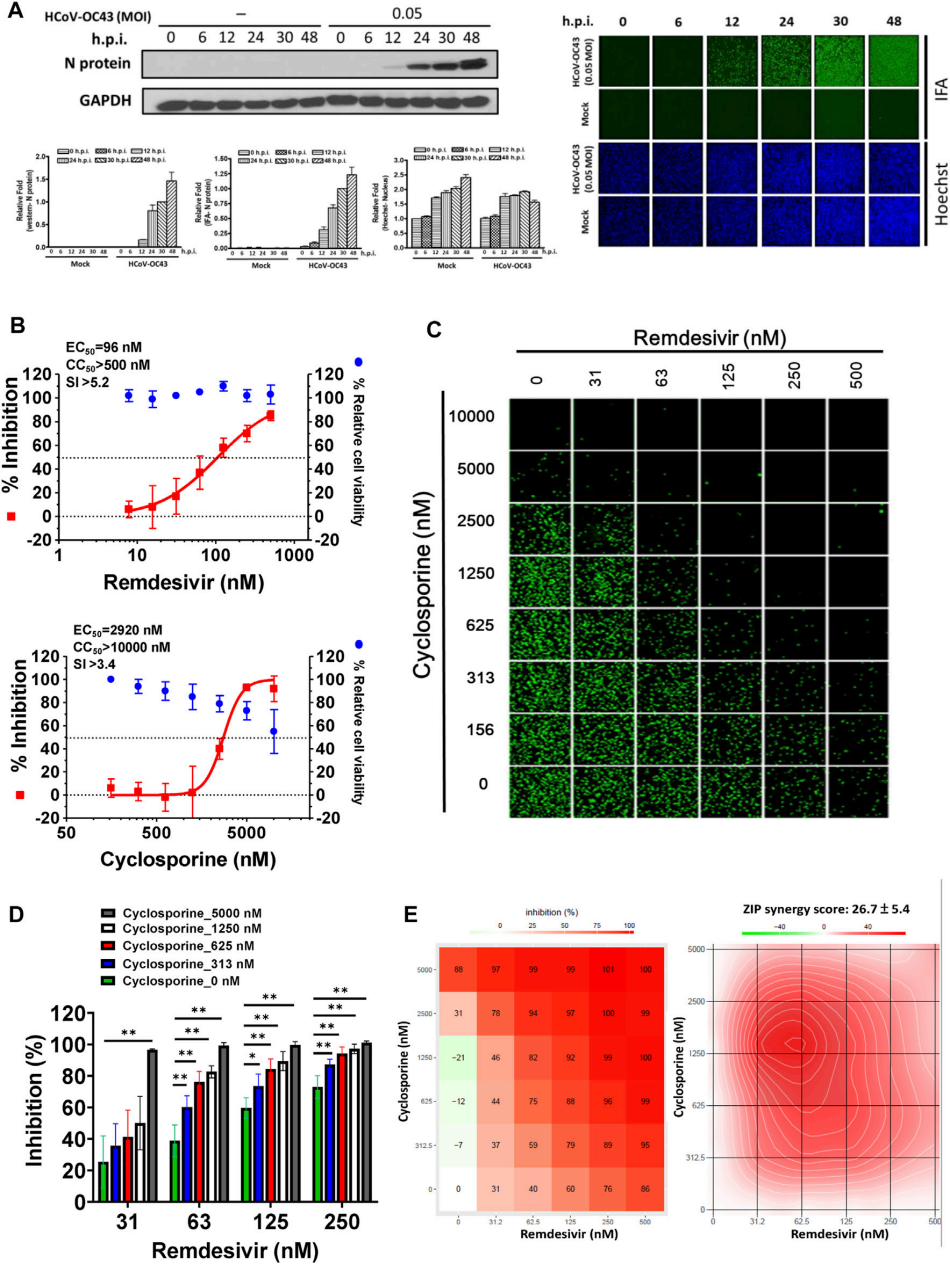
Single or combined treatment of remdesivir and cyclosporine profoundly reduced HCoV-OC43 infection as assayed by immunofluorescence (IFAs) in human HCT-8 colorectal carcinoma cells. **(A)** Time course of HCoV-OC43 N protein expression in infected HCT-8 cells. Western analysis and IFA were performed against an antibody against HCoV-OC43 N protein (Mab9013) with the samples at the indicated time points. **(B)** Single treatments of remdesivir and cyclosporine reduced HCoV-OC43 infection in HCT-8 cells in a dose dependent manner. **(C–E)** Combined treatments of remdesivir and cyclosporine synergistically reduced the HCoV-OC43 infection in HCT-8 cells. IFAs were performed with an antibody against N protein (green) of HCoV-OC43 in HCoV-OC43 (0.05 MOI) infected HCT-8 cells at 30 h.p.i. treated with vehicle (0.5% DMSO) or compounds as indicated. Nuclei (blue) were counter stained with Hoechst dye and used to determine the relative cell viability by using the number of nuclei in vehicle control as 100% (Supplementary Figure S1). The fluorescent signal was normalized with cell viability to calculate the infection rate that no compound treatment was set at 100%. HCT-8 cells were seeded the day before compound treatment or HCoV-OC43 infection. The tested compounds were added to the wells 1 h prior to the addition of HCoV-OC43 at an MOI of 0.05 and the resulting cultures incubated for an additional 30 h at 37°C. AVE ±SD of three independent experiments are shown **(A, B, D)** The statistical significance was evaluated using one-way ANOVA followed by Tukey’s multiple comparison test. * and ** denote statistical significance of *p* < 0.05, and *p* < 0.01 respectively. IFA images shown are representative of three independent experiments **(C)**. Shown inhibition % (AVE) and synergy scores (AVE ± SD) are from three independent experiments **(E)** analyzed *via* SynergyFinder (https://synergyfinder.fimm.fi/).

**FIGURE 2:**
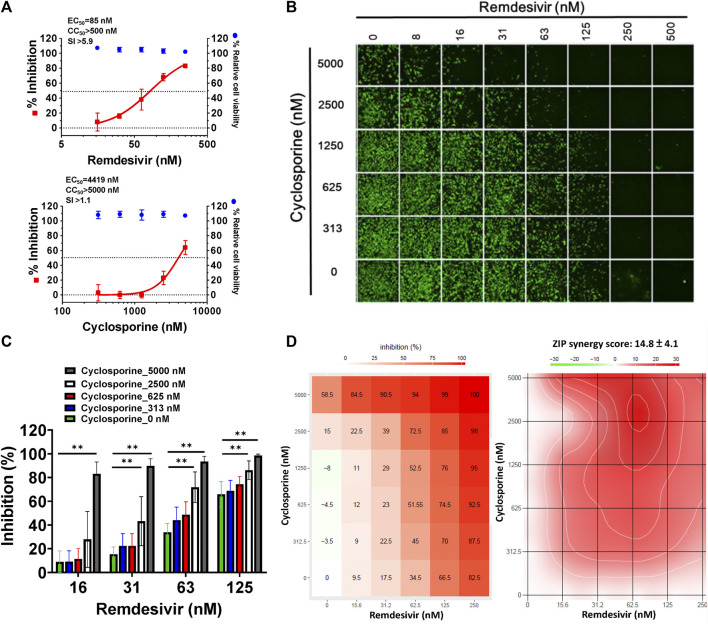
Single or combined treatments of remdesivir and cyclosporine profoundly reduced HCoV-OC43 infection in human fetal lung fibroblast MRC-5 cells assayed by IFA. **(A)** Single treatments of remdesivir and cyclosporine reduced HCoV-OC43 infection in MRC-5 cells in a dose dependent manner. **(B–D)** Combined treatments of remdesivir and cyclosporine synergistically reduced the HCoV-OC43 infection in MRC-5 cells. IFAs were performed with an antibody against N protein (green) of HCoV-OC43 in HCoV-OC43 (0.05 MOI) infected MRC-5 cells at 30 h.p.i. treated with vehicle (0.5% DMSO) or compounds as indicated. Nuclei (blue) were counter stained with Hoechst dye and used to determine the relative cell viability by using the number of nuclei in vehicle control as 100% (Supplementary Figure S2). The fluorescent signal of IFA was normalized with cell viability to calculate the infection rate that no compound treatment was set at 100%. MRC-5 cells were seeded the day before compound treatment or HCoV-OC43 infection. Tested compounds were added to the wells 1 h prior to the addition of HCoV-OC43 at an MOI of 0.05. The resulting cultures were then incubated for an additional 30 h at 37°C. AVE ± SD of three independent experiments are shown **(A and C)**. The statistical significance was evaluated using one-way ANOVA followed by Tukey’s multiple comparison test. * and ** denote statistical significance of *p* < 0.05, and *p* < 0.01 respectively. IFA images shown are representative of three independent experiments **(B)**. Shown inhibition % (AVE) and synergy scores (AVE ± SD) are from three independent experiments **(D)** analyzed *via* SynergyFinder (https://synergyfinder.fimm.fi/).

As shown in Figure 1A, the replication of OC43 in infected HCT-8 cells rose with time over a period of 48 h as examined by western analysis and IFA. To investigate the anti-coronaviral effects of remdesivir and cyclosporine, human colorectal carcinoma cells HCT-8 and human fetal fibroblast cells MRC-5 were infected by HCoV-OC43 at an MOI of 0.05 and incubated at various concentrations of the indicated compounds for 30 h. As shown in Figures 1B, 2A and Table 1, remdesivir and cyclosporine inhibited HCoV-OC43 in a dose dependent manner with EC_50_ values of 96 ± 34 nM and 2,920 ± 364 nM in HCT-8 cells and 85 ± 23 nM and 4,419 ± 490 nM in human MRC-5 fetal lung fibroblast cells, respectively.

**TABLE 1:** Antiviral activities and IL-6 reduction of remdesivir and cyclosporine against HCoV-OC43 and SARS-CoV-2. Shown are AVE ± SD from three independent experiments.

CoV	Cell line	Assay	EC50 (nM)	
			Remdesivir	Cyclosporine
HCoV-OC43	HCT-8	Viral activity by IFA	96 ± 34	2,920 ± 364
HCoV-OC43	MRC-5	Viral activity by IFA	85 ± 23	4,419 ± 490
SARS-CoV-2	Vero E6	Viral activity by IFA	3,962 ± 303	7,213 ± 143
SARS-CoV-2	Vero E6	Viral plaque formation	291 ± 91	6,767 ± 1827
HCoV-OC43	MRC-5	IL-6 by ELISA	224 ± 53	1,292 ± 352

The combined effects of remdesivir and cyclosporine against HCoV-OC43 were also investigated at various concentrations of each (Figures 1C, 2B). The results revealed that these two drugs exerted a significantly synergistic effect when administered in combination (Figures 1D, 2C), with synergy scores of 26.7 ± 5.4 and 14.8 ± 4.1, and most synergistic area scores of 43.1 and 19.8 against HCoV-OC43 in HCT-8 and MRC-5 cells respectively, as assayed by IFA (Figures 1C, 2B) and analyzed by SynergyFinder (Figures 1E, 2D; Table 2).

**TABLE 2:** The synergistic inhibition of HCoV-OC43, SARS-CoV-2, and IL-6 production by remdesivir and cyclosporine. Synergy scores were calculated and analyzed by SynergyFinder, ZIP method. Shown synergy scores are AVE ± SD from three independent experiments.

CoV	Cell line	Assay	Synergy score	Most synergistic area score
HCoV-OC43	HCT-8	Viral activity by IFA	26.7 ± 5.4	43.1
HCoV-OC43	MRC-5	Viral activity by IFA	14.8 ± 4.1	19.8
SARS-CoV-2	Vero E6	Viral activity by IFA	25.0 ± 2.5	65.5
SARS-CoV-2	Vero E6	Viral plaque formation	43.7 ± 14.9	46.2
HCoV-OC43	MRC-5	IL-6 by ELISA	13.0 ± 5.7	25.8

### Inhibitory Effects of Remdesivir and Cyclosporine, Alone and in Combination, on IL-6 Cytokine Production in HCoV-OC43 Infected Human MRC-5 Fetal Lung Fibroblast Cells

**FIGURE 3:**
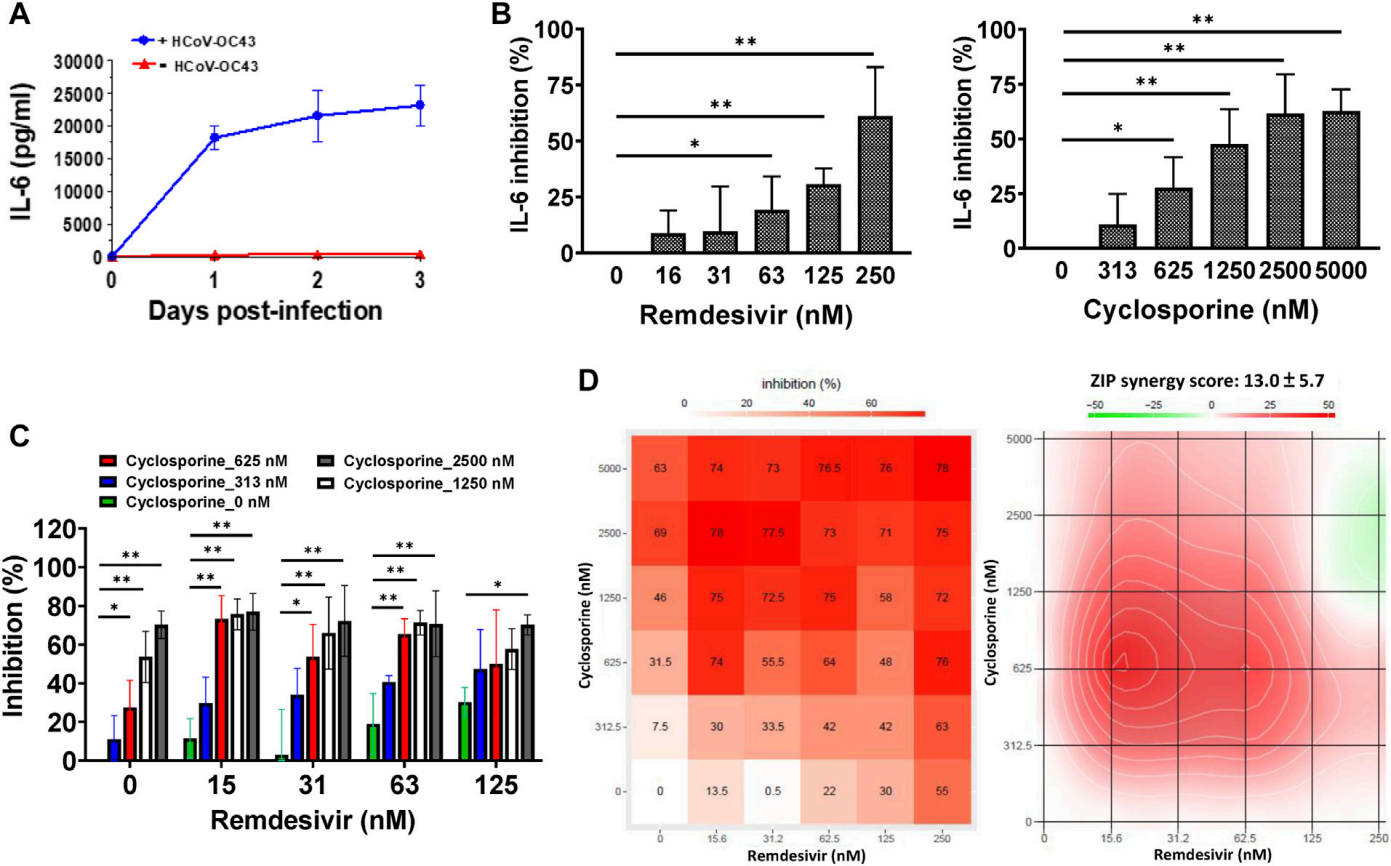
Reduction of IL-6 levels by single and combined treatments of remdesivir and cyclosporine in HCoV-OC43 infected human MRC-5 fetal lung fibroblast cells. **(A)** IL-6 production induced by HCoV-OC43 infection in MRC-5 cells over 3 days. **(B)** Dose dependent reduction of IL-6 levels by remdesivir and cyclosporine treatments in HCoV-OC43 infected MRC-5 cells at 30 h.p.i. Shown are the AVE ± SD from three independent experiments **(A–C)** The two-tailed unpaired Student’s t test was used evaluated the dose effect of single drug treatment on IL-6 production. **(C and D)** Synergistic reduction of IL-6 levels produced by HCoV-OC43 infected MRC-5 cells treatment with the combination of remdesivir and cyclosporine. MRC-5 cells were seeded the day before compound treatment or HCoV-OC43 infection. The tested compounds were added to the wells 1 h prior to the addition of HCoV-OC43 at an MOI of 0.05. The resulting cultures were then incubated for an additional 30 h **(B–D)**, or as indicated **(A)**, at 37°C. Subsequently, the culture supernatants were subjected to detection and quantitation of IL-6 by ELISA. AVE ± SD from three independent experiments **(A–C)** are shown; The statistical significance was evaluated using one-way ANOVA followed by Tukey’s multiple comparison test. * and ** denote statistical significance of *p* < 0.05, and *p* < 0.01 respectively **(B and C)**. Shown inhibition % (AVE) and synergy scores (AVE ± SD) are from three independent experiments **(D)** analyzed *via* SynergyFinder (https://synergyfinder.fimm.fi/).

Since IL-6 is a pivotal biomarker in COVID-19 disease progression (Henry et al., 2020; McElvaney et al., 2020), we examined IL-6 levels in HCoV-OC43-infected and -uninfected HCT-8 and MRC-5 cells. Greater than 20,000 pg/ml of IL-6 was found to be produced by HCoV-OC43-infected MRC-5 cells over the course of 3 days (Figure 3A); IL-6 levels in uninfected MRC-5 cells were very low (Figure 3A; Table 3). However, IL-6 was not detected in the culture supernatants of HCoV-OC43-infected or uninfected HCT-8 cells (Table 3). Therefore, the effects of remdesivir and cyclosporine on IL-6 cytokine production were examined in HCoV-OC43-infected MRC-5 cells at 30 h.p.i. over a time period equivalent to the time needed for the HCoV-OC43-infected human MRC-5 fetal lung fibroblast cells to produce 18,186 ± 1,895 pg/ml of IL-6 (Table 3).

**TABLE 3:** IL-6 production in human colorectal carcinoma HCT-8 and human fetal lung fibroblast MRC-5 cells with or without HCoV-OC43 infection at 30 h.p.i. Shown are AVE ± SD from three independent experiments.

Cell line	HCoV-OC43 infection	IL-6 quantitative range
HCT-8	−	<minimum 1.5 pg/ml
HCT-8	+	minimum 1.5 pg/ml
MRC-5	−	303 ± 66 pg/ml
MRC-5	+	18,186 ± 1,895 pg/ml

As shown in Figure 3B and Table 1, remdesivir and cyclosporine separately reduced IL-6 production in MRC-5 cells infected with HCoV-OC43 at 30 h.p.i. in a dose dependent manner, with EC_50_ values of 224 ± 53 nM and 1,292 ± 352 nM, respectively. Similarly, the combined effects of remdesivir and cyclosporine on HCoV-OC43-induced IL-6 production were investigated at varying concentrations of each. The results showed that these two drugs exerted a significantly synergistic reduction of IL-6 production by HCoV-OC43-infected MRC-5 cells when administered in combination (Figure 3C), with a synergy score of 13.0 ± 5.7 and a most synergistic area score of 25.8, as assayed by ELISA and analyzed by SynergyFinder (Figure 3D; Table 2).

### Inhibitory Effects of Remdesivir and Cyclosporine, Alone and in Combination, on SARS-CoV-2 in Vero E6 Cells

We further examined whether the combination of remdesivir and cyclosporine could synergistically inhibit SARS-CoV-2. Two disparate assays (IFA and plaque formation) were performed to examine the single and combined inhibitory effects of remdesivir and cyclosporine on SARS-CoV-2 in Vero E6 cells.

**FIGURE 4:**
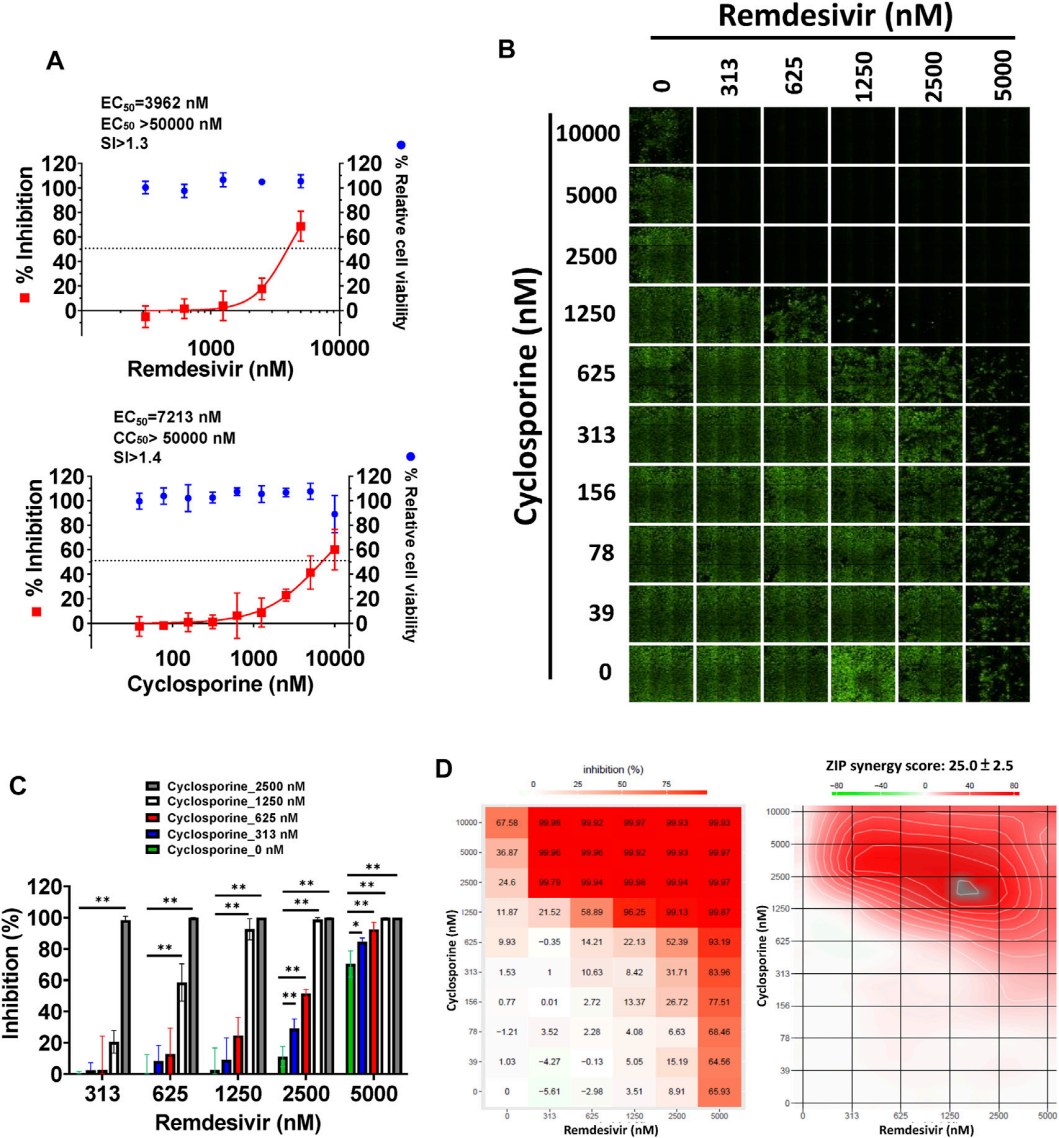
Single or combined treatments of remdesivir and cyclosporine profoundly reduced SARS-CoV-2 infection of Vero E6 cells assayed by IFA. **(A)** Single treatments of remdesivir and cyclosporine reduced SARS-CoV-2 infection of Vero E6 cells in a dose dependent manner. **(B–D)** Combined treatments of remdesivir and cyclosporine synergistically reduced SARS-CoV-2 infection in Vero E6 cells. IFAs were performed with antibody against SARS-CoV-2 N (green) and DAPI staining (blue) for the Vero E6 host live cells. Vero E6 cells were treated with each compound at the indicated concentrations for 1 h at 37°C. The cells were adsorbed with SARS-CoV-2 (TCDC#4) at MOI = 0.01 for 1 h at 37°C. After virus adsorption, the cells were washed with PBS and fresh medium with each compound added at the indicated concentrations and then incubated for 1 day. The cells were fixed with 4% paraformaldehyde and permeabilized with 0.5% Triton X-100. The cells were stained with anti-SARS-CoV-2 N protein antibody and anti-human IgG-Alexa Fluor 488 (green). Nuclei were counter stained with DAPI (blue) and used to determine the relative cell viability by using the number of nuclei in vehicle control as 100% (Supplementary Figure S3). N protein expression was measured using a high-content image analysis system (Molecular Devices). The fluorescent signal was normalized with cell viability to calculate the infection rate that no compound treatment was set at 100%. EC_50_ and CC_50_ values were calculated by Prism software. Shown are the AVE ± SD from three independent experiments **(A and C)** * and ** denote statistical significance of *p* < 0.05, and *p* < 0.01 respectively. Shown results of IFA are representative of three independent experiments **(B)**. Shown inhibition % (AVE) and synergy scores (AVE ± SD) are from three independent experiments **(D)** analyzed *via* SynergyFinder (https://synergyfinder.fimm.fi/).

As shown in Figure 4A and Table 1, remdesivir and cyclosporine exhibited dose-dependent SARS-CoV-2 inhibition with respective EC_50_ values of, 3,962 ± 303 nM and 7,213 ± 143 nM in Vero E6 cells at 1 day post-infection (d.p.i.) with an antibody against SARS-CoV-2 N protein. The combined effects of remdesivir and cyclosporine against SARS-CoV-2 were then investigated at various concentrations of each. The results revealed that the inhibitory effects of these two drugs against SARS-CoV-2 in Vero E6 cells, as assayed by IFA at 1 d.p.i. (Figure 4B), were significantly synergistic (Figure 4C) with a synergy score of 25.0 ± 2.5 (analyzed by SynergyFinder) and a most synergistic area score of 65.5 (Figure 4D and Table 2).

**FIGURE 5:**
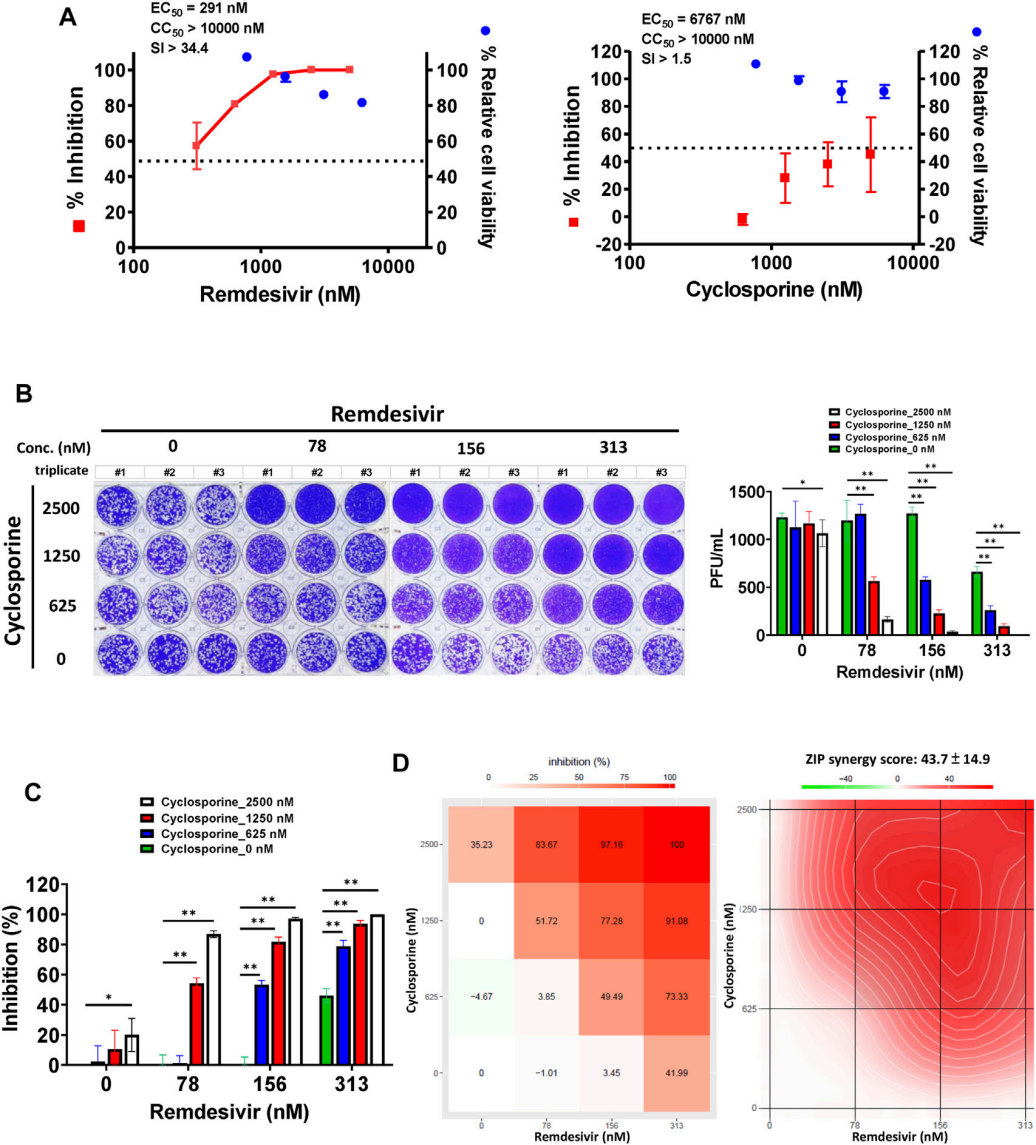
Single or combined treatment with remdesivir and cyclosporine synergistically reduced infectious SARS-CoV-2 viral loads as determined by plaque formation assays. **(A)** Single treatment of SARS-CoV-2-infected Vero E6 cells with remdesivir and cyclosporine reduced the number of plaque-forming units in a dose dependent manner. **(B)** Combined treatment of SARS-CoV-2-infected Vero E6 cells with remdesivir and cyclosporine significantly decreased plaque formation caused by SARS-CoV-2. **(C and D)** Combined treatment of SARS-CoV-2-infected Vero E6 cells with remdesivir and cyclosporine synergistically reduced SARS-CoV-2 viral loads. Plaque assays were performed in triplicate using 24-well tissue culture plates. Vero E6 cells were seeded in DMEM with 10% FBS and antibiotics 1 day before infection. SARS-CoV-2 was added to the cell monolayer for 1 h at 37°C. Subsequently, viruses were removed and the cell monolayer was washed once with PBS before covering with overlay media containing 1% methylcellulose (Sigma, cat#M0387) and the test compounds at indicated concentrations for 5–7 days. The cells were fixed with 10% formaldehyde solution (Marcon^TM^ Chemicals, cat #H121-08) overnight. After removal of overlay media, the cells were stained with crystal violet and the plaques were counted. The percentage of inhibition was calculated as [1–(VD/VC)] × 100%, where VD and VC refer to the virus titer in the presence and absence of the test compound, respectively. The inhibition in plaque formation results shown are representative of three independent experiments, each in triplicate **(B)**. Cell viability was determined as described (Kuo et al., 2021) by measurement of relative alkaline phosphatase activity; viability in the absence of compound treatment was set as 100%. AVE ± SD of three independent experiments were shown **(A and C)**; The statistical significance was evaluated using one-way ANOVA followed by Tukey’s multiple comparison test. * and ** were used to denote the statistical significance for *p* < 0.05, and *p* < 0.01 respectively **(C)**. Shown inhibition % (AVE) and synergy scores (AVE ± SD) are from three independent experiments **(D)** analyzed *via* SynergyFinder (https://synergyfinder.fimm.fi/).

A plaque formation assay was also undertaken, to examine the combined effect of remdesivir and cyclosporine on the SARS-CoV-2 infectious dose at 5–7 d.p.i. in Vero E6 cells (Figure 5). The plaque formation assay is traditionally used to determine the infectious virus dose–the number of plaque-forming units in a virus sample. Plaque formation in culture plates of SARS-CoV-2-infected confluent Vero E6 cells took 5–7 days. Plaques were counted manually and the numbers corrected based on the sizes of plaques as described in Methods. Remdesivir and cyclosporine were again found to separately inhibit SARS-CoV-2 in a dose dependent manner with respective EC_50_ values of 291 ± 91 nM and 6,767 ± 1,827 nM (Figure 5A; Table 1). Their combined inhibitory effect was found to be significantly synergistic as shown in Figures 5B,C, with a synergy score of 43.7 ± 14.9 and a most synergistic area score of 46.2 analyzed by SynergyFinder (Figure 5D; Table 2).

## Conclusion and Discussions

Remdesivir is a prodrug which is metabolized into a nucleoside triphosphate that irreversibly bonds with the RdRp of SARS-CoV-2, blocking viral transcription after cell entry (Shannon et al., 2020; Hanafin et al., 2021). It is used clinically for the treatment of COVID-19 patients, but its efficacy is limited (Beigel et al., 2020; Goldman et al., 2020; Spinner et al., 2020) and its mechanism of action does not address the cytokine storm that also accompanies the disease progression of COVID-19 (McElvaney et al., 2020). Blockade of key pathogenic cytokines in COVID-19 patients should ameliorate disease progression and exert viral inhibition. Elevated IL-6 levels in COVID-19 patients play an important role in disease progression and severity (Henry et al., 2020; McElvaney et al., 2020), and the IL-6 inhibitor tocilizumab is a potential alternative therapy for COVID-19 patients experiencing a cytokine storm (Klopfenstein et al., 2020; Toniati et al., 2020; Xu et al., 2020). However, more controlled clinical trials are needed to confirm its efficacy and safety (Campochiaro et al., 2020; Lan et al., 2020; Stone et al., 2020).

Simultaneous reduction of coronaviral loads and mitigation of the cytokine storm may constitute an efficacious treatment of severe COVID-19. Herein, we identified the immunosuppressant cyclosporine by screening for inhibitors of HCoV-OC43, and found that it exerted a significantly synergistic inhibitory effect against HCoV-OC43 and SARS-CoV-2 in combination with remdesivir (Figures 1, 2, 4, 5; Tables 1, 2). These drugs also reduced coronavirus IL-6 production synergistically, when used in combination (Figure 3; Tables 2, 3). Whereas cyclosporine reduces IL-6 *via* down-regulation of its production (Stephanou et al., 1992; Iacono et al., 1997) (which contributes to its anti-viral activity), remdesivir targets coronaviral RdRp (Gordon et al., 2020; Pruijssers et al., 2020) to reduce viral loads and thereby reducing IL-6 production. In combination, these drugs are capable of profoundly reducing coronaviral loads and IL-6 production in a synergistic manner. Furthermore, the doses of cyclosporine (3.6–4.8 mg/kg) which correspond to the measured EC_50_ values (3–4 μM) or lower, when in combination with remdesivir, are actually lower than those in current clinical use (∼15 mg/kg for the initial dose and 5–10 mg/kg for the maintenance doses), which result in cyclosporine plasma concentrations of 250–1000 ng/ml (Novais Sandimmune®, https://www.rxlist.com/sandimmune-drug.htm#side_effects). Similarly, the EC_50_ values of remdesivir herein are also equivalent or lower than those in current clinical use (100–200 mg) (Grein et al., 2020). Thus, the cyclosporine and remdesivir levels measured in this study are of clinical significance.

The synergy of the combinations of remdesivir with itraconazole (an anti-fungal), fluoxetine (Schloer et al., 2021) (an anti-depressant), or calcium-channel blocker diltiazem (Pizzorno et al., 2020) respectively was reported, but the effects were only marginal [synergy scores ∼12 analyzed by SynergyFinder (Pizzorno et al., 2020; Schloer et al., 2021)]. On the other hand, Janus kinases (JAKs) play a critical role in the cytokine storm of severe COVID-19 patients (Luo et al., 2020) and the combined treatment of remdesivir with the JAK inhibitor baricitinib was found to further reduce recovery time and improve the clinical status of severe COVID-19 patients compared to remdesivir alone (Kalil et al., 2020). Therefore, the synergistic effect of the combined treatment of remdesivir and cyclosporine in reducing coronaviral load and IL-6 levels identified herein merits further study for application in moderately or severely ill COVID-19-patients whose SARS-CoV-2 infection is complexed with a cytokine storm, as well as other applicable conditions identified in the future.

These results validate our strategy of treating COVID-19 with two drugs–one to address viral load, and the other cellular factors. The search for other combined treatments for patients with different progressive and pathogenic stages of COVID-19 merit further exploration.

## Data Availability

The original contributions presented in the study are included in the article/Supplementary Material, further inquiries can be directed to the corresponding author.
