# Supplementary material for: Remdesivir and Cyclosporine Synergistically Inhibit the Human Coronaviruses OC43 and SARS-CoV-2
Source: Front Pharmacol. 2021 Aug 13;12:706901. doi: 10.3389/fphar.2021.706901 (PMC8409573; doi:10.3389/fphar.2021.706901)

# Supplementary Information

**Remdesivir and cyclosporine synergistically inhibit the human coronaviruses OC43 and SARS-CoV-2**

Hsing-Yu Hsu[1#], Cheng-Wei Yang[1#], Yue-Zhi Lee[1#], Yi-Ling Lin[2#], Sui-Yuan Chang[3#], Ruey-Bing Yang[2], Jian-Jong Liang[2], Tai-Ling Chao[3], Chun-Che Liao[2], Han-Chieh Kao[3], Szu-Huei Wu[1], Jang-Yang Chang[1], Huey-Kang Sytwu[4], Chiung-Tong Chen[1], Shiow-Ju Lee[1]*

[1]Institute of Biotechnology and Pharmaceutical Research, National Health Research Institutes, Miaoli, Taiwan, ROC.
[2]Institute of Biomedical Sciences, Academia Sinica, Taipei, Taiwan, ROC.
[3]Institute of Clinical Laboratory Sciences and Medical Biotechnology, College of Medicine, National Taiwan University, Taipei, Taiwan, ROC.
[4]National Institute of Infectious Diseases and Vaccinology, National Health Research Institutes, Miaoli, Taiwan, ROC.

*To whom correspondence should be addressed. * (S.-J. L.) Tel: +886-37-206166 ext. 35715; Fax: +886-37-586456; Email: slee@nhri.org.tw

# Authors with equal contribution

## Supplementary Figures

**Supplementary Figure 1 :**
Cell viability in combined treatments of remdesivir and cyclosporine in human HCT-8 colorectal carcinoma cells as assayed in Figure 1C. Shown are AVE ± S.D. of three independent experiments.

**Supplementary Figure 2 :**
Cell viability in combined treatments of remdesivir and cyclosporine in human fetal lung fibroblast MRC-5 cells as assayed in Figure 2B. Shown are AVE ± S.D. of three independent experiments.

**Supplementary Figure 3 :**
Cell viability in combined treatments of remdesivir and cyclosporine in Vero E6 cells cells as assayed in Figure 4B. Shown are AVE ± S.D. of three independent experiments.

**Supplementary Figure 1 :**

Cell viability in combined treatments of remdesivir and cyclosporine in human HCT-8 colorectal carcinoma cells as assayed in Figure 1C. Shown are AVE ± S.D. of three independent experiments. Figure 1C (IFA) was also shown here for clarity.

**Cell Viability (%)**

| Cyclosporine (nM) \ Remdesivir (nM) | 0 | 31 | 63 | 125 | 250 | 500 |
|---|---|---|---|---|---|---|
| 10000 | 55±19% | 58±18% | 59±16% | 60±16% | 56±19% | 54±17% |
| 5000 | 73±8% | 77±8% | 78±5% | 81±6% | 76±10% | 72±11% |
| 2500 | 79±7% | 81±12% | 87±6% | 85±6% | 84±10% | 76±7% |
| 1250 | 85±11% | 88±10% | 89±12% | 91±11% | 89±10% | 85±11% |
| 625 | 90±8% | 92±10% | 96±10% | 97±9% | 97±7% | 93±9% |
| 313 | 94±6% | 101±8% | 98±15% | 100±10% | 101±10% | 95±8% |
| 156 | 100±3% | 101±7% | 104±7% | 106±7% | 106±7% | 102±7% |
| 0 | 102±3% | 103±4% | 106±1% | 108±3% | 104±4% | 101±4% |

**Figure 1C (IFA)**

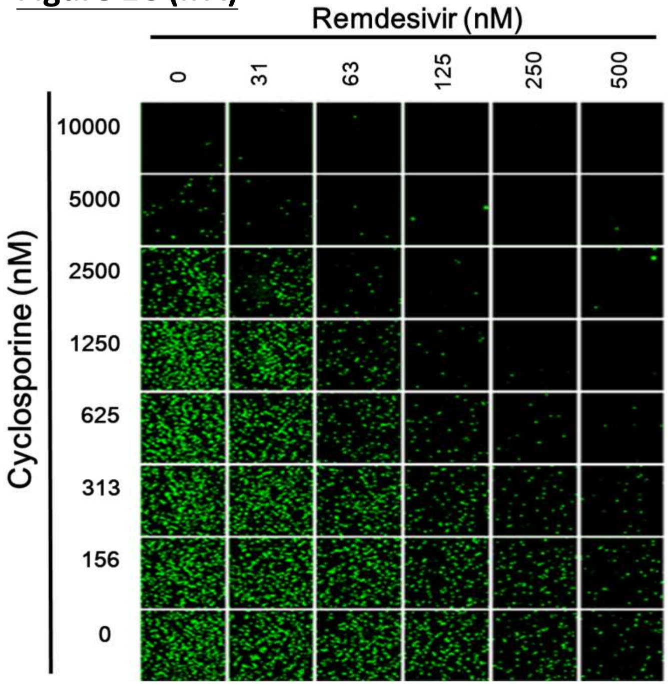

**Supplementary Figure 2 :**

Cell viability in combined treatments of remdesivir and cyclosporine in human fetal lung fibroblast MRC-5 cells as assayed in Figure 2B. Shown are AVE ± S.D. of three independent experiments. Figure 2B (IFA) was also shown here for clarity.

**<u>Cell Viability (%)</u>**

| Cyclosporine (nM) \ Remdesivir (nM) | 0 | 8 | 16 | 31 | 63 | 125 | 250 | 500 |
|---|---|---|---|---|---|---|---|---|
| 5000 | 107±2% | 108±4% | 105±4% | 106±3% | 106±6% | 107±4% | 106±5% | 106±4% |
| 2500 | 109±4% | 108±3% | 109±5% | 108±5% | 107±3% | 108±6% | 105±7% | 107±4% |
| 1250 | 108±7% | 109±6% | 111±6% | 112±4% | 112±3% | 110±3% | 107±6% | 108±4% |
| 625 | 108±4% | 109±4% | 109±5% | 110±5% | 108±6% | 107±4% | 106±3% | 108±5% |
| 313 | 107±5% | 108±4% | 108±2% | 107±4% | 107±4% | 105±1% | 107±4% | 105±3% |
| 0 | 105±5% | 105±1% | 107±2% | 105±3% | 105±4% | 103±3% | 105±5% | 103±4% |

**<u>Figure 2B (IFA)</u>**

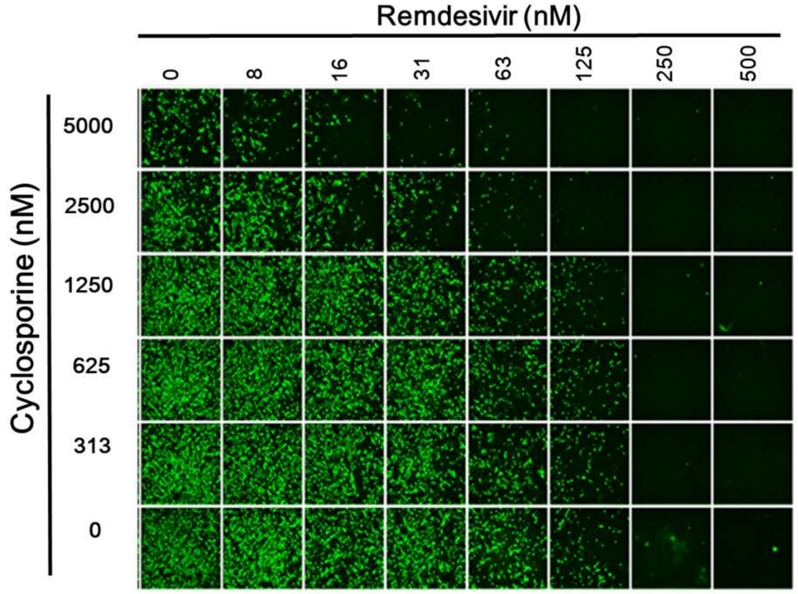

**Supplementary Figure 3 :**

Cell viability in combined treatments of remdesivir and cyclosporine in Vero E6 cells as assayed in Figure 4B. Shown are AVE ± S.D. of three independent experiments. Figure 4B (IFA) was also shown here for clarity.

**Cell Viability (%)**

| Cyclosporine (nM) \ Remdesivir (nM) | 0 | 313 | 650 | 1250 | 2500 | 5000 |
|---|---|---|---|---|---|---|
| 10000 | 84±10% | 90±12% | 86±11% | 87±14% | 90±11% | 89±12% |
| 5000 | 105±6% | 110±6% | 110±6% | 112±7% | 111±3% | 108±5% |
| 2500 | 108±6% | 115±4% | 116±5% | 112±2% | 116±4% | 107±3% |
| 1250 | 116±7% | 118±8% | 116±8% | 112±11% | 108±3% | 105±6% |
| 650 | 110±8% | 112±8% | 109±3% | 110±4% | 106±5% | 107±3% |
| 313 | 110±4% | 108±5% | 107±7% | 103±5% | 102±4% | 103±4% |
| 156 | 107±3% | 107±7% | 109±7% | 104±4% | 100±6% | 102±9% |
| 78 | 107±2% | 104±2% | 105±6% | 101±4% | 103±4% | 104±5% |
| 39 | 106±4% | 105±2% | 104±4% | 99±4% | 97±4% | 100±5% |
| 0 | 105±4% | 105±1% | 107±5% | 98±4% | 100±4% | 100±0% |

**Figure 4B (IFA)**

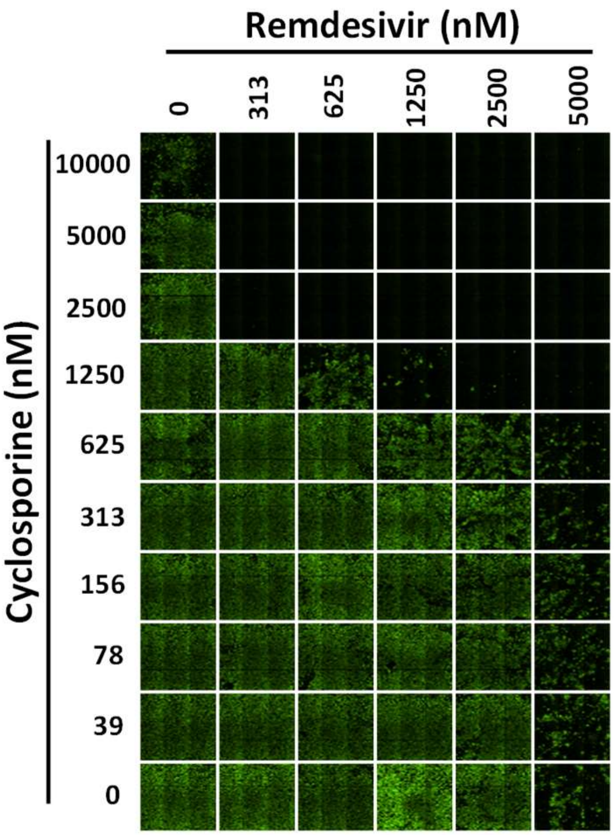

Supplement: Supplementary file 1 [file DataSheet1.PDF]
